# What evidence exists on the impact of climate change on some of the worst invasive fish and shellfish? A systematic map protocol

**DOI:** 10.1186/s13750-022-00273-z

**Published:** 2022-05-21

**Authors:** Mohamad Nor Azra, Mohd Iqbal Mohd Noor, Yeong Yik Sung, Elizabeth R. Lawrence, Mazlan Abd Ghaffar

**Affiliations:** 1 Institute of Marine Biotechnology, Universiti Malaysia Terengganu (UMT), 21030 Kuala Nerus, Terengganu Malaysia; 2 Climate Change Adaptation Laboratory, Institute of Marine Biotechnology (IMB), Universiti Malaysia Terengganu (UMT), 21030 Kuala Nerus, Terengganu Malaysia; 3 Faculty of Business Management, Universiti Teknologi MARA (UiTM) (Pahang), 27600 Raub, Pahang Malaysia; 4 Institute for Biodiversity and Sustainable Development, Universiti Teknologi MARA (UiTM), 40450 Shah Alam, Selangor Malaysia; 5 Quebec Centre for Biodiversity Science, Concordia University, 1455 Boulevard de Maisonneuve O, Montréal, QC H3G 1M8 Canada; 6 Faculty of Science and Marine Environment, Universiti Malaysia Terengganu, 21030 Kuala Nerus, Terengganu Malaysia; 7 Higher Institution Centre of Excellence (HICoE), Institute of Tropical Aquaculture and Fisheries (AKUATROP), Universiti Malaysia Terengganu, 21030 Kuala Nerus, Terengganu Malaysia

**Keywords:** Biodiversity loss, Exotic, Introduced, Environmental monitoring, Non-native, 100 world’s worst, Climate change

## Abstract

**Background:**

The Intergovernmental Science-Policy Platform on Biodiversity and Ecosystem Services (IPBES) has estimated that invasive alien species (IAS) might cause billions of dollars of losses every year across the world. One example is South-East Asia, where IAS have caused an estimated loss of 33.5 billion USD, affecting the environment, human health, and agricultural production. Factors associated with climate change, such as increased carbon dioxide (CO_2_), heavy precipitation, and elevated temperatures is expected to facilitate biological invasion, leading only to further financial and public health loss. Thus, further study is needed to identify, collate and categorise what evidence exists on the impacts of climate change on fish and shellfish species that contribute to the list of “One Hundred of the World’s Worst Invasive Alien Species” as identified by the International Union for Conservation of Nature’s (IUCN). Such mapping will identify regions more at risk of biological invasion as climate change progresses.

**Methods:**

We outline a systematic mapping review protocol that follows the Guideline and Standards for Evidence Synthesis in Environmental Management and RepOrting standards for Systematic Evidence Syntheses (ROSES). We describe how peer-reviewed articles will be collected from Web of Science and Scopus, and then analyzed to create knowledge maps on the impact climate change has on invasive species. Finally, we speculate on how our results will aid future management of invasive species in the light of climate change.

**Supplementary Information:**

The online version contains supplementary material available at 10.1186/s13750-022-00273-z.

## Background

To date, the total number of invasive alien species (IAS) has reached an estimated record of 17,000 species worldwide, causing billions of dollars in losses each year [1]. For example, in South-East Asia alone, IAS are estimated to cause 33.5 billion USD worth of damages. IAS can cause financial loss through several different ways, but one factor is through the loss of local biodiversity, largely through direct or indirect competition. This loss represents a serious problem internationally, negatively affecting not only biodiversity, but also human and animal health, as well as agricultural and/or fisheries production. Although there are many policies and regulations focusing on some of the worst IAS, the management practices and implementation must still be improved. Additionally, while there is ample evidence for the direct detrimental and often irreversible effects that IAS have on terrestrial, freshwater, and marine ecosystems, there lacks sufficient knowledge regarding the impact IAS have on ecosystem services. This lack of knowledge hinders the conservation and sustainable use of biodiversity.

Previous studies found that IAS and climate change constitute two global threats to biodiversity that may act synergistically [2, 3] but also may interact in other ways [4]. Impacts associated with climate change, such as increased carbon dioxide (CO_2_) concentration, heavy precipitation [5], or elevated temperatures [6] may facilitate biological invasion. This will likely lead to an increase in frequency and abundance of IAS across the globe, necessitating international and national efforts to mitigate issues related to invasive alien species. Some such groups include the Intergovernmental Science-Policy Platform on Biodiversity and Ecosystem Services (IPBES), International Union for Conservation of Nature’s (IUCN), Invasive Species Specialist Group (ISSG), Invasive Species Compendium (ISC), and the National Committee on IAS of Malaysia (as well as national committees in other countries). There also already exist many examples of international policy related to invasive species such as the Aichi Target (Target 9), Convention on Biological Diversity (Nagoya Protocol), United Nations Sustainable Development Goals (SDG)—Goal 15.8 (Life on Land), and various national policies in different countries (e.g. Action Plan for Aquatic Invasive Alien Species in Malaysia) [7]. All these different organizations and policies demonstrate the importance of managing IAS and their impacts.

Management of IAS can be improved in many ways [7–9]. First, improving the education and public awareness about IAS would facilitate local prevention. Such education would include research on all aspects of the management, pathways of introduction, ecological impacts, containment technologies, future threats and problems, prevention or control, and mitigation efforts. Second, conducting a risk assessment on all potential IAS before they are introduced would help to set a formal and rigorous response plan, as well as ensuring there is the capacity to contain and eradicate any potential IAS. Finally, strengthening quarantine inspection and enforcement at entry points as well as international borders would help reduce risk of introduction. Such measures could include enforcement legislation, the implementation of safeguards against water-based IAS, establishment of quarantine facilities, and an effective monitoring/implementation action plan. A systematic study could help better understanding on the goal of supporting evidence-informed decision making in conservation and environmental management for various stakeholders in the future [10–14].

One global initiative to improve awareness of IAS was established in 2000 by the IUCN working group of ISSG, which established the “One Hundred of the World’s Worst Invasive Alien Species” list [15]. Here, a species is listed based on two main criteria: (i) the severity of their impact on biological diversity and/or human activities, and (ii) their ability to illustrate important issues surrounding biological invasion [16]. Focusing on species of fish and shellfish, there are nine and four species, respectively, listed in the worldwide Global Invasive Species Database (GISD). These species include the following:

A. Green crab, *Carcinus maenas*
B. Walking catfish, *Clarias batrachus*
C. Common Carp, *Cyprinus carpio*
D. Zebra mussel, *Dreissena polymorpha*
E. Chinese mitten crab, *Eriocheir sinensis*
F. Western mosquito fish, *Gambusia affinis*
G. Nile perch, *Lates niloticus*
H. Largemouth bass, *Micropterus salmoides*
I. Mediterranean mussel, *Mytilus galloprovincialis*
J. Rainbow trout, *Oncorhynchus mykiss*
K. Mozambique tilapia, *Oreochromis mossambicus*
L. Marine clam, *Potamocorbula amurensis*
M. Brown trout, *Salmo trutta*

### Stakeholder engagement

Because our systematic map will inform a climate change related program and project undertaken by Universiti Malaysia Terengganu (UMT), we worked with project partners (i.e. researchers, government agencies, and NGOs in Malaysia) to develop our methodological strategies and objectives. The aim of the Malaysian research program is to increase climate resilience for flora and fauna, especially invasive species in freshwater and marine areas impacted by the climate change while supporting community livelihoods, food security, health, and well-being. In our discussions, stakeholders provided topic-relevant input by refining the scope of the map question, suggesting search terms for the search strategy, and suggested relevant studies and sources of grey literature. Stakeholders will also provide comment on other parts of the systematic map as it progresses, for example, the appropriateness of the meta-data extraction.

### Objective of the systematic map

The primary objective for our systematic map is to identify, collate and categorise how climate change impacts on some of the worst invasive fish and shellfish in the world. We focus on the 13 species listed above and how different elements of climate change will impact the type of invasiveness (e.g. spread, establishment, etc.) of said species. We aim to demonstrate global trends in the literature and identify areas in research that could be improved.

### Primary question

What evidence exists on the impact of climate change on some of the worst invasive fish and shellfish.

#### Definitions of the questions

For simplicity, we broke our research objective down into a “Population–Exposure–Outcome” structure, where “Population” represents any of the 13 world’s worst fish and shellfish IAS, “Exposure” represents aspects of climate change and “Outcome: represent the type of invasiveness.

## Methods

This systematic mapping review protocol applies the Guideline and Standards for Evidence Synthesis in Environmental Management (version 5.0) [17] and conforms to ROSES reporting standards [18] (Additional file 1).

### Search strategy

#### Bibliographic databases

Literature search for the systematic mapping review will be undertaken using the Science Citation Index Expanded (SCI-EXPANDED), part of the Web of Science Core Collection (Web of Science) and Scopus. Databases are made available by Universiti Malaysia Terengganu. In Web of Science, the search will be run based on the “topic” (TS) field, which includes article titles, abstracts, keywords, and “KeyWords Plus” (automatically generated terms pulled from the titles of cited articles). In Scopus, article titles, abstracts, and keywords will be searched using the same search string as in Web of Science, outlined below. All years of data will be included. The searches and search results will be conducted in English due to the scope, timeline, and funding of this project. Search results will then be exported into a CSV-format, as well as into the reference management software Endnote.

#### Supplemental searching methods

##### Google Scholar search

A Google Scholar (www.scholar.google.co.uk) search will be done using the Advanced search functionality. We will restrict our search to exclude patents, as well as ensure that search terms are searched “anywhere in article”. We will use the Publish or Perish software to collate and download the first 1000 search results, then add them to the bibliographic database constructed from the Web of Science and Scopus searches.

##### Organizational websites and online catalogues

Twenty-seven relevant organizational websites and topical catalogues will be used to search for additional literature. Search strings for these sources were drawn from the database search string and adapted to the search capabilities of each website. The websites are listed in Box 1, and details of each search string used are in Additional file 2.

**Table:** Box 1. List of websites to search for relevant studies

• https://www.invasivespeciescentre.ca/invasive-species/what-is-at-risk/climate-change/. • https://www.invasivespeciesinfo.gov/subject/climate-change. • https://invasives.org.au/our-work/climate-change/climate-change-invasive-species/. • https://toolkit.climate.gov/topics/ecosystem-vulnerability/invasive-species. • https://www.worldwildlife.org/magazine/issues/fall-2015/articles/animals-affected-by-climate-change. • https://www.ecolandscaping.org/. • https://climateadaptationexplorer.org/. • https://pi-casc.soest.hawaii.edu/. • https://www.cabi.org/about-cabi/climate-change/. • https://www.sprep.org/. • https://www.noaa.gov/. • https://www.eea.europa.eu/. • https://wildlife.org/. • https://www.unep.org/. • https://www.ipcc.ch/. • https://www.iucn.org/. • http://www.issg.org/. • https://www.ipbes. • http://elabs.ebd.csic.es/web/invasibes/home. • https://www.risccnetwork.org/. • https://www.un.org/sustainabledevelopment/. • https://www.the-scientist.com/. • https://ec.europa.eu/environment/index_en. • https://research.csiro.au/climate/. • https://www.conservation.org/. • https://www.gbif.org/. • https://www.nature.org/en-us/.	

### Search terms

A well-constructed search string is key for a quality systematic mapping review [19]. A good search string will retrieve the most studies related to the review objectives. To build our keyword search string, a scoping exercise was first conducted using Web of Science to ensure any relevant literature would not be overlooked. The scoping exercise used a list of ten articles as a benchmark (Additional file 3), and we tested different search strings until one that identified all ten articles was found. This resulted in the final search string with the highest efficiency, as shown in Additional file 2. As we conduct our mapping exercises, improvement of search terms will be done based on the scoping exercise to make sure no relevant literature will be overlooked [18].

As our primary objective focuses on the impact on aquatic IAS towards climate change, all Population and Exposure terms will be included in the search string. We use both common names and scientific names for the IAS. No comparator and/or outcome component will be added as both criteria will be determined during data coding. Additionally, we will not restrict our search based on timeframe or document type.

#### Population terms: Selected Alien invasive species

TS = ((“carcinus maenas”) OR (“european green crab”) OR (“european shore crab”) OR (“green crab”) OR (“shore crab”) OR (“clarias batrachus”) OR (“clarias catfish”) OR (“climbing perch”) OR (“freshwater catfish”) OR (“thailand catfish”) OR (“walking catfish”) OR (“cyprinus carpio”) OR (“common carp”) OR (“dreissena polymorpha”) OR (“eurasian zebra mussel”) OR (“moule zebra”) OR (“wandering mussel”) OR (“zebra mussel”) OR (“eriocheir sinensis”) OR (“chinese freshwater edible crab”) OR (“chinese mitten crab”) OR (“chinese river crab”) OR (“shanghai crab”) OR (“gambusia affinis”) OR (“live-bearing tooth-carp”) OR (“live bearing tooth carp”) OR (“mosquito fish”) OR (“western mosquitofish”) OR (“lates niloticus”) OR (“nile perch”) OR (“victoria perch”) OR (“micropterus salmoides”) OR (“black bass”) OR (“green bass”) OR (“green trout”) OR (“largemouth bass”) OR (“largemouth black bass”) OR (“mytilus galloprovincialis”) OR (“bay mussel”) OR (“blue mussel”) OR (“mediterranean mussel”) OR (“oncorhynchus mykiss”) OR (“baja california rainbow trout”) OR (“brown trout”) OR (“coast angle trout”) OR (“coast range trout) OR (“rainbow trout”) OR (“redband trout”) OR (salmon trout) OR (“silver trout”) OR (“steelhead trout”) OR (“summer salmon”) OR (“oreochromis mossambicus”) OR (“common tilapia”) OR (“java tilapia”) OR (“mozambique cichlid”) OR (“mozambique mouth breeder”) OR (“mozambique mouthbrooder”) OR (“mozambique tilapia”) OR (tilapia) OR (“potamocorbula amurensis”) OR (“suspension feeding clam”) OR (“suspension-feeding clam”) OR (“asian bivalve”) OR (“asian clam”) OR (“brackish-water corbula”) OR (brackish water corbula”) OR (“chinese clam”) OR (“marine clam”) OR (“salmo trutta”) OR (“brook trout”) OR (“brown trout”) OR (“orange fin”) OR (“orkney sea trout”) OR (“salmon trout”) OR (“salmo trota”) OR (“sea trout”) OR (“trout”) OR (“whiting”) OR (“whitling”)).

#### Exposure terms: climate change

TS = ((climat*) OR (“global warm*”) OR (“seasonal* variat*”) OR (“extrem* event*”) OR (“environment* variab*”) OR (“anthropogenic effect*”) OR (“stres*”) OR (“greenhouse effect*”) OR (“sea level ris*”) OR (erosio*) OR (“agricult* runoff”) OR (“weather* variab*”) OR (“weather* extrem*”) OR (“extreme* climat*”) OR (“environment* impact*”) OR (“environment* chang*”) OR (“anthropogenic stres*”) OR (“temperature ris*”) OR (“temperature effect*”) OR (“warm* ocean”) OR (“sea surface* temperat*”) OR (heatwav*) OR (acidific*) OR (hurrican*) OR (el nino) OR (“el nino”) OR (“la nina”) OR (la-nina) OR (drought*) OR (flood*) OR (“high precipit*”) OR (“heavy rainfall*”) OR (“CO_2_ concentrat*”) OR (“melt* of the glacier*”) OR (“melt* ice*”) OR (“therm* stress*”) OR (drought) OR (hypoxia)).

The focus of this systematic map is on the impact of climate change on aquatic IAS. Our search terms included:

TS = ((“*Selected Alien invasive species*”) AND (“Climate Change”)).

### Eligibility criteria

#### Inclusion criteria

- Populations
  We will report on the 13 species of invasive shellfish and fish included in “100 worst world fish and shellfish species” from the Global Invasive Species Databases (GISD). No other organisms will be considered. If a study includes results for both focus species and non-focus species, this will not be included in the systematic map.
- Exposures
  We also only include literature that focuses on direct climate variables and their derivatives. Studies must have focused on the effects of climate change by measuring the direct effect of climate variables on an invasive species’ population. Such climate variables include all those listed in “Exposure terms: climate change” section.
- Comparators
  No comparator is required for this map.
- Outcomes
  We will only include studies that focus on the impacts of climate change on the twenty type of invasiveness criteria of (i) World Distribution, (ii) Local distribution, (iii) Prey/Host Range, (iv) Predator range, (v) Economic value of Hosts, (vi) Economic value of species, (vii) Reproductive performance, (viii) Life Cycle, (ix) Climatic tolerance, (x) Detectability, (xi) Competition with native species, (xii) Potential of Introduction, (xiii) Potential of Establishment, (xiv) Potential of Spread, (xv) Control Measures, (xvi) Economic impact (negative impact), (xvii) Economic impact (positive impact), (xviii) Environmental impact, (xix) Heritage/Infrastructure damage and (xx) Impact on Human Health. Studies will be accepted if they report measures of these response, which could be represented by statistical data. Those studies reporting only the effects on other outcomes will be excluded. Where outcomes are only modeled or predicted, they are not eligible.

#### Other inclusion criteria

- Context/setting
  We focus our review on studies performed in aquatic settings. However, we do not limit the geographical extent and instead any studies across the globe are eligible.
- Studies and publications
  Literature to be included will report empirical research published in peer-reviewed journals or conference proceedings and grey literature. This includes experimental, quasi-experimental, non-experimental, narrative, and observational studies. Furthermore, included literature will provide analysis of the results of the exposure.

#### Exclusion criteria

- Populations and exposure
  Our review will exclude populations of invasive aquatic plants, single-cell organisms, phytoplankton, zooplankton, or coral reef invasive species. We will also exclude literature that investigates issues not related to climate change, such as anthropogenic habitat destruction, construction waste runoff, bauxite residues runoff, or flood mitigation.
- Type of context/setting
  Our review will exclude literature that reports on exposure in terrestrial or other non-aquatic environments.
- Studies and publications
  We will exclude any study focusing on life cycle assessment, prediction- or forecasting-based assessment(s), laboratory experiments of indirect impacts, or review articles. Literature that explores the effects of changes in secondary variables without linking those changes to direct variables will also be excluded. Any study that is not original, or published in a language other than English will also be excluded. The reason to choose English language is not about questioning the impact on Non-English research but to reduce potential bias from reviewers due to limited understanding of other languages. Including studies published in non-English languages also may increase costs, time, and expertise in non-English languages.
  Finally, studies that report case studies, cohort studies, cross-sectional research designs, conference abstracts without a corresponding full-length peer-reviewed paper, and unpublished research (e.g., unpublished dissertations/theses) will be excluded.

### Article screening

All articles identified using the search string provided in “Search terms” section will be uploaded in EndNote X7, where duplicate studies will be removed. After duplicates are removed, screening will begin by evaluating based on (i) title and abstract eligibility and (ii) full text eligibility. Articles will then be assessed using eligibility criteria listed in “Eligibility criteria” section. Articles that pass the inclusion criteria and articles that have uncertain relevance at the title and abstract stage will be included and reviewed at the full text level. As screening progresses, we will record the inclusion/exclusion status of articles for each stage. The reasoning for excluding articles at the full-text stage will be recorded in a additional file.

The screening process will be conducted by two reviewers. Ten percent of the literature retrieved will be selected and screened by each of the reviewers independently at both title and abstract and full text stages. Consistency between decisions will be analyzed using the Cohen’s Kappa test. Kappa values > 0.60 are considered significant for consistent and accurate decisions between individuals [20, 21]. In addition, any disagreements between reviewers will be discussed and resolved regardless of Kappa value results. In cases where a publication that was authored or co-authored by any of the two reviewers selected, the publication will be referred to another reviewer in the team for assessment.

### Study validity assessment

The critical appraisal of study validity and strength is not carried out for this mapping exercise as the intention of the map is not to examine the robustness of the study designs. However, data coding strategy will include information regarding the design of each study. The data produced could be used further by users of this systematic map to assess the evidence presented in the article.

### Data coding strategy

The coding for descriptive analysis includes basic information such as: title of publication, citation, year of publication, and location of lead author’s affiliated institution country. Then, a narrative synthesis will be conducted using a thematic category that identifies each article’s key results. These categories mainly describe a study’s hypotheses, and explain the general impact of climate change on aquatic ecosystems. We follow a two-step coding process where first a line-by-line review is done on the articles to identify the key impacts on IAS. A code label is then assigned to each article to cluster all information into a common theme. Two of the reviewers will independently perform the coding process; however, we discuss between all reviewers to ensure a robust set of common themes.

## Study mapping, presentation, and implications

Our final systematic mapping review will include summary figures and tables of illustrating study characteristics for all data collected. In addition, heatmaps will be produced which will cross tabulate two variables (e.g. type of climate change-related measure and type of outcome) and detail the volume of evidence (number of studies) within each cell of the table. The review will highlight the direct effects of climate change issues on multiple effect it has on the species (such as chemical changes, physiological changes, and behavioral changes). We expect our review to provide valuable insight for future research recommendations regarding invasive species, as we will identify important knowledge gaps (un- or under-represented subtopics warranting further research, or clusters (well-represented subtopics that are amenable to full synthesis by a systematic review). Based on our results, recommendations will be made for prioritization for future research and mitigation of climate change impact. Finally, our full database containing extracted information will be made readily available for download for future use.

## Supplementary Information

**Additional file 1.** ROSES form.

**Additional file 2.** Search strategy.

**Additional file 3.** Benchmark article.

## Data Availability

Not applicable.
