# Supplementary material for: What evidence exists on the impact of climate change on some of the worst invasive fish and shellfish? A systematic map protocol
Source: Environ Evid. 2022 May 21;11:19. doi: 10.1186/s13750-022-00273-z (PMC11378826; doi:10.1186/s13750-022-00273-z)
Supplement: Supplementary file 2 — Additional file 2. Search strategy. [file 13750_2022_273_MOESM2_ESM.docx]

**Additional File 2: Evolution of search string.**

**Synergistic impacts of climate change on world ‘s worst fish and shellfish invasive alien species: A systematic map protocol**

**Additional File**

This document summarizes the main steps taken to identify search terms and develop the search string through scoping exercises.

**Scoping**

An extensive set of keywords was compiled using expert knowledge, relevant journal articles and books and by consulting with the stakeholder group members. The keywords were combined to develop a search string relevant to the following predefined elements generated by the primary question:

Population: 13 fish and shellfish invasive alien species

Exposure: Climate Change

A scoping exercise was conducted using Web of Science Core Collections to modify the search terms by examining for specificity and sensitivity of alternate terms, wildcards and Boolean operators. The asterisk was used as a wildcard to allow for singular or plural words to be identified in the same search. A list of alternative terms was established with the aid of a thesaurus and screening of relevant citations.

Various iterations of the search string were tested against a ‘benchmark’ list of 10 key articles selected to represent a range of academic studies focus on climate change and invasive’s species, covering a range of authors, journals, and research topics relevant to the scope of the question. Each test search was carefully recorded as a percentage of the test list and saved for access later. The search was finalized once all the studies in the ‘benchmark’ list were found.

The final search string was composed of terms related to the ‘Population’ and ‘Exposure’ elements. The basic terms relating to the ‘Population’ elements of invasive species were too broad and resulted in a large number of irrelevant articles. Those keywords that resulted in only a few extra articles were not included, and those that resulted in at least 50 more articles were included. The search term for invasive species is from Global Invasive Species Databases (GISD)..

To deal with specific exposure phrase, this article uses three way of search strings ranging from restrictive to broadest approach. The three-specific way of search are: (i) using quotes (e.g. “climate change”) ; (ii) using the OR operator and (iii) using the AND operator (e.g. (climate AND “species”). Before accepting the final search string, an exercise was conducted to review the potentially relevant articles that are excluded when using the single term “climate”. The titles and abstracts of the first 100 articles in the search result were reviewed briefly to identify relevant articles. Only 5% of the articles were deemed moderately relevant to the study, therefore the final search string was approved. Additionally, the search will be limited to articles published from 2000 onwards in order to focus the study on the last 2 decade which has seen a noticeable increase in research that integrates climate changes datasets to better understand focus of study.

| **Step** | **Focus** | **Search type** | **Search string** | **Number of publications retrieved in WOS CC** | **Comprehensiveness**  **(Based on 20 articles indexed in WOS CC) *** | **Comments** |
| --- | --- | --- | --- | --- | --- | --- |
| **1** | Population | TS | TS=((“carcinus maenas”) OR (“clarias batrachus”) OR (“cyprinus carpio”) OR (“dreissena polymorpha”) OR (“eriocheir sinensis”) OR (“gambusia affinis”) OR (“lates niloticus”) OR (“micropterus salmoides”) OR (“mytilus galloprovincialis”) OR (“oncorhynchus mykiss”) OR (“oreochromis mossambicus”) OR (“potamocorbula amurensis”)) | **60,661** | **7/10**  **(70%)** | **The inclusion only scientific keyword did not bring sufficient article retrieve** |
| **2** |  |  | TS=((“european green crab”) OR (“european shore crab”) OR (“green crab”) OR (“shore crab”) OR (“clarias catfish”) OR (“climbing perch”) OR (“freshwater catfish”) OR (“thailand catfish”) OR (“walking catfish”) OR (carp) OR (“eurasian zebra mussel”) OR (“moule zebra”) OR (“wandering mussel”) OR (“zebra mussel”) OR (“chinese freshwater edible crab”) OR (“chinese mitten crab”) OR (“chinese river crab”) OR (“shanghai crab”) OR (“live bearing tooth$carp”) OR (“live bearing tooth carp”) OR (“mosquito fish”) OR (“western mosquitofish”) OR (“nile perch”) OR (“victoria perch”) OR (“black bass”) OR (“green bass”) OR (“green trout”) OR (“largemouth bass”) OR (“largemouth black bass”) OR (“bay mussel”) OR (“blue mussel”) OR (“mediterranean mussel”) OR (“baja california rainbow trout”) OR (“brown trout”) OR (“coast angle trout”) OR (“coast range trout) OR (“rainbow trout”) OR (redband) OR (salmon trout) OR (“silver trout”) OR (steelhead) OR (“summer salmon”) OR (“common tilapia”) OR (“java tilapia”) OR (“mozambique cichlid”) OR (“mozambique mouth breeder”) OR (“mozambique mouthbrooder”) OR (“mozambique tilapia”) OR (tilapia) OR (“suspension feeding clam”) OR (“suspension$feeding clam”) OR (“asian bivalve”) OR (“asian clam”) OR (“brackish$water corbula”) OR (brackish water corbula”) OR (“chinese clam”) OR (“marine clam”) OR (“salmo trutta”) OR (blacktail) OR (“brook trout”) OR (“brown trout”) OR (“galway sea trout”) OR (“orange fin”) OR (“orkney sea trout”) OR (“salmon trout”) OR (“salmo trota”) OR (“sea trout”) OR (trout) OR (whiting) OR (whitling)) | **114,763** | **7/10**  **(70%)** | **The inclusion only common name keyword did not bring sufficient article retrieve** |
| **3** |  |  | TS=((“carcinus maenas”) OR (“european green crab”) OR (“european shore crab”) OR (“green crab”) OR (“shore crab”) OR (“clarias batrachus”) OR (“clarias catfish”) OR (“climbing perch”) OR (“freshwater catfish”) OR (“thailand catfish”) OR (“walking catfish”) OR (“cyprinus carpio”) OR (carp) OR (“dreissena polymorpha”) OR (“eurasian zebra mussel”) OR (“moule zebra”) OR (“wandering mussel”) OR (“zebra mussel”) OR (“eriocheir sinensis”) OR (“chinese freshwater edible crab”) OR (“chinese mitten crab”) OR (“chinese river crab”) OR (“shanghai crab”) OR (“gambusia affinis”) OR (“live bearing tooth$carp”) OR (“live bearing tooth carp”) OR (“mosquito fish”) OR (“western mosquitofish”) OR (“lates niloticus”) OR (“nile perch”) OR (“victoria perch”) OR (“micropterus salmoides”) OR (“black bass”) OR (“green bass”) OR (“green trout”) OR (“largemouth bass”) OR (”largemouth black bass”) OR (“mytilus galloprovincialis”) OR (“bay mussel”) OR (“blue mussel”) OR (“mediterranean mussel”) OR (“oncorhynchus mykiss”) OR (“baja california rainbow trout”) OR (“brown trout”) OR (“coast angle trout”) OR (“coast range trout) OR (“rainbow trout”) OR (redband) OR (salmon trout) OR (“silver trout”) OR (steelhead) OR (“summer salmon”) OR (“oreochromis mossambicus”) OR (“common tilapia”) OR (“java tilapia”) OR (“mozambique cichlid”) OR (“mozambique mouth breeder”) OR (“mozambique mouthbrooder”) OR (“mozambique tilapia”) OR (tilapia) OR (“potamocorbula amurensis”) OR (“suspension feeding clam”) OR (“suspension$feeding clam”) OR (“asian bivalve”) OR (“asian clam”) OR (“brackish$water corbula”) OR (brackish water corbula”) OR (“chinese clam”) OR (“marine clam”) OR (“salmo trutta”) OR (blacktail) OR (“brook trout”) OR (“brown trout”) OR (“galway sea trout”) OR (“orange fin”) OR (“orkney sea trout”) OR (“salmon trout”) OR (“salmo trota”) OR (“sea trout”) OR (trout) OR (whiting) OR (whitling)) | **128, 229** | 10/10  (100%) | Full population block built. We are satisfied that our population block is able to capture the maximum of literature: comprehensiveness 100%. |
| 2 | *Population + Exposure* | TS | **POPULATION AND** ((climat*) | 3,183 | 5/10  (50%) | To reduce the search hits. We added a context. However, this obviously reduced the comprehensiveness. |
|  |  | TS | **POPULATION AND** ((climat*) OR (“global warm*”) OR (“seasonal* variat*”) OR (“extrem* event*”) OR (“environment* variab*”) OR (“anthropogenic effect*”) OR (“multiple stres*”) OR (“greenhouse effect*”) OR (“sea level ris*”) OR (erosio*) OR (“agricult* runoff”) OR (“weather* variab*”) OR (“weather* extrem*”) OR (“extreme* climat*”) OR (“environment* impact*”) OR (“environment* chang*”) OR (“anthropogenic stres*”) OR (“temperature ris*”) OR (“temperature effect*”) OR (“warm* ocean”) OR (“sea surface* temperat*”) OR (heatwav*) OR (acidific*) OR (hurrican*) OR (el$nino) OR (“el nino”) OR (“la nina”) OR (la$nina) OR (drought*) OR (flood*) OR (“high precipit*”) OR (“heavy rainfall*”) OR (“CO_2_ concentrat*”) OR (“melt* of the glacier*”) OR (“melt* ice*”)) | 8,775 | 10/10  (100%) | Full exposure block built. We are satisfied that our exposure block is able to capture the maximum of literature: comprehensiveness 100%. This is our chosen search string: acceptable number of search hits, maximum comprehensiveness, and appropriate combination of key words responding to the project objectives and the stakeholders’ demands. |
