# Supplementary material for: What evidence exists on the impact of climate change on some of the worst invasive fish and shellfish? A systematic map protocol
Source: Environ Evid. 2022 May 21;11:19. doi: 10.1186/s13750-022-00273-z (PMC11378826; doi:10.1186/s13750-022-00273-z)
Supplement: Supplementary file 3 — Additional file 3. Benchmark article. [file 13750_2022_273_MOESM3_ESM.docx]

**Additional file 3.** Articles used for benchmark studies.

| Title | Author | Journal |
| --- | --- | --- |
| Biogeographic vulnerability to ocean acidification and warming in a marine bivalve | (Van Colen, Jansson, Saunier, Lacoue-Labathe, & Vincx, 2018) | Marine Pollution Bulletin |
| Measuring the effects of temperature rise on Mediterranean shellfish aquaculture | (Martinez et al., 2018) | Ecological Indicators |
| Adapt, move or die – how will tropical coral reef fishes cope with ocean warming | (Habary, Johansen, Nay, Steffensen, & Rummer, 2017) | Global Change Biology |
| Global warming of salmon and trout rivers in the northwestern US: Road to ruin or Path Through Purgatory? | (Isaak et al., 2018) | Transactions of the American Fisheries Society |
| Acute Effects of Different Temperature in the Blood Parameters of Common Carp (Cyprinus Carpio) | (Bozorgnia, Hosseinifard, & Alimohammadi, 2011) | 2^nd^ International Conference on Environmental Science and Technology |
| Climate Change and Alien Species in South Africa | (Irlich, Richardson, Davies, & Chown, 2014) | Invasive Species and Global Climate Change |
| The potential distribution of zebra mussels in the United States | (Drake & Bossenbroek, 2004) | Bioscience |
| Possible Effects of Sea Level Rise on Suitable Nursery Habitats of Largemouth Bass (Micropterus salmoides) in Tidal Freshwater | (Love, 2015) | American Midland Naturalist |
| The impact of climate change on the parasites and infectious diseases of aquatic animals | (Marcogliese, 2008) | OIE Revue Scientifique et Technique |
| Impacts of salicylic acid in Mytilus galloprovincialis exposed to warming conditions | (Freitas et al., 2020) | Environmental Toxicology and Pharmacology |
